# Editorial: The role of immune and stromal mediators in the formation of pre-metastatic and metastatic niches: the gateway to metastasis

**DOI:** 10.3389/fimmu.2026.1904388

**Published:** 2026-06-18

**Authors:** Michal A. Rahat, Dalit Barkan, Adit Ben-Baruch

**Affiliations:** 1 Department of Immunology, Ruth and Bruce Rappaport Faculty of Medicine, Technion-Israel Institute of Technology, Haifa, Israel and the Carmel Medical Center, Haifa, Israel; 2 The Paul and Herta Amir School of Medicine, Department of Human Biology, University of Haifa, Haifa, Israel; 3 The Shmunis School of Biomedicine and Cancer Research, George S. Wise Faculty of Life Sciences, Tel Aviv University, Tel Aviv, Israel

**Keywords:** extracellular matrix (ECM), immune/inflammatory cells, metastasis, metastatic niche (MN), premetastatic niche (PMN), stromal cells

## 1 Introduction

Current treatments such as surgery, chemotherapy, and immunotherapy have improved survival in many cancers, yet metastasis remains the main clinical challenge because it is often treatment-resistant, detected late, and systemic (1–3). Disseminating tumor cells (DTCs) can leave the primary tumor, seed distant organs, and remain dormant, causing recurrence months or years after tumor removal. Recent work has clarified mechanisms governing migration, colonization, dormancy, and pre-metastatic (PMN) and metastatic niche (MN) formation (4, 5). These processes are regulated by tumor cell intrinsic programs (6), as well as by interactions between the cancer cells tumor microenvironment components, including stromal cells, extracellular matrix (ECM) (7, 8) and immune/inflammatory cells (9, 10). This Research Topic highlights recent advances in defining these interactions and their therapeutic implications.

## 2 Stromal-immune coordination and organ-specific niche dynamics

The overview by Zhai et al. describes how stromal mediators and immune cells orchestrate the PMN and its transition into a MN. Primary tumors condition distant tissues through soluble factors and extracellular vesicles (EVs) that recruit immune cells, activate stromal cells, and remodel the ECM. Immunosuppressive populations, including regulatory T cells (Tregs), myeloid-derived suppressor cells (MDSCs), tumor-associated macrophages (TAMs), and N2-polarized neutrophils, cooperate with cancer-associated fibroblasts (CAFs) and endothelial cells to create the immunosuppressive, ECM-remodeled environment of PMN and MN. These interactions also influence whether DTCs remain dormant or resume growth, underscoring the need for combination therapies that block metastatic progression.

PMN formation is organ-specific, shaped by tissue structure, resident cells, ECM composition, and soluble mediators. Mourad et al. illustrates this in colorectal cancer (CRC), where liver metastases exhibit a tolerogenic, immunosuppressive microenvironment associated with resistance to immune checkpoint inhibitors (ICIs). In contrast, the lung is more immune-reactive, with stronger lymphocyte activation and better outcomes. The review also discusses combining immunotherapy with anti-angiogenic or targeted therapies to overcome organ-specific resistance.

## 3 Cellular contributors to niche formation and immune evasion

Locatelli et al. review how platelets and platelet-derived extracellular vesicles (PEVs) contribute to PMN and MN formation. Beyond coagulation, platelets maintain vascular integrity, regulate inflammation, and support angiogenesis. In cancer, tumor-derived factors alter platelets and PEV cargo, enabling delivery of pro-tumoral signals across tissues. Platelets shield circulating tumor cells from immune surveillance, especially by natural killer (NK) cells, promote endothelial arrest through selectins and integrins, and release mediators that enhance angiogenesis, epithelial-to-mesenchymal transitions (EMT), seeding, and myeloid cell polarization. Transfer of CD41 from PEVs to tumor cells activates MAPK and AKT signaling, upregulates matrix metalloproteinases (MMPs), and promotes ECM remodeling. Together, these findings position platelets as coordinators of angiogenesis, immune evasion, and ECM dynamics in metastasis, although standardized isolation methods and translational studies are still needed.

Oricco et al. examine the bone PMN and show that osteoclasts (OCs) directly suppress the anti-tumor activity of NK cells, enabling non-small cell lung cancer stem cells to evade immune recognition and expand in bone. In co-culture, IL-15-stimulated NK cells limit OC survival by inducing apoptosis, whereas OCs reduce activating receptors such as DNAM-1 and NKp44 and increase inhibitory checkpoints, particularly TIGIT and TIM-3, on NK cells. This impaired NK-cell degranulation and tumor-cell killing identify OCs as active immune modulators in the bone niche and TIGIT as a promising therapeutic target.

## 4 Examples of ECM-mediated metastatic support

Xia et al. show in orthotopic and transgenic mouse models of breast cancer that metastatic lungs contain markedly lower levels of nidogen-1, a fibroblast-derived basement-membrane protein. In a melanoma experimental metastasis model, nidogen-1 knockdown increased lung metastasis and disrupted alveolar structure, with fragmented endothelium, poorly defined basement membrane, and enlarged interstitial spaces. These findings suggest that nidogen-1 preserves basement-membrane integrity and acts as a barrier to metastatic colonization, whereas its loss facilitates metastatic lesion formation.

Xin et al. analyzed single-cell and bulk RNA-sequencing data from gastric cancer (GC) and GC with peritoneal metastasis, identifying elevated expression of immunosuppressive genes, including the CAF-derived ECM protein periostin (POSTN). CAFs expressed high levels of POSTN, whereas GC cells expressed little. POSTN-overexpressing fibroblasts promoted CD8+ T-cell exhaustion, increased PD-1 expression, and enhanced peritoneal dissemination *in vivo.* Mechanistically, POSTN increased tumor cell adhesion and spheroid formation while suppressing antitumor immunity. Combined inhibition of POSTN signaling (Cilengitide) and immune checkpoints reduced T-cell exhaustion and metastatic spread, highlighting the role of POSTN in immune evasion and metastasis.

## 5 Chemokine and CD147-driven signaling in the formation of PMN and MN

Tian et al. review the CXCL12/CXCR4 axis as a central driver of tumor progression, organ-specific metastasis, and immune evasion in CRC. CRC cells with high CXCR4 migrate toward organs such as the liver and lungs, where CXCL12 is abundant. This signaling promotes a large variety of metastasis-enhancing process and reshapes the tumor microenvironment (TME) by recruiting immunosuppressive populations while limiting immune-effector entry. The authors further describe a CXCL12-dependent mechanism in which binding to keratin-19 on tumor cells forms a barrier-like structure that impairs infiltration by CD8+ T cells and NK cells. Inhibitors of this pathway, including Plerixafor (AMD3100), LY2510924, and Peptide R, reduce invasion, EMT, and chemoresistance in preclinical models, supporting combinations with ICIs in metastatic CRC.

In a review paper, Su et al. describe CD147/EMMPRIN, a membrane glycoprotein overexpressed in many malignancies, as a central hub driving cancer metabolism, immune evasion, and PMN formation. CD147 can be released in EVs or shed as a soluble protein, thus influencing distant organs. Through interactions with multiple partners, including itself, it activates pathways that support proliferation, EMT, migration, matrix degradation, angiogenesis, drug resistance, immune modulation, and metabolic reprogramming. Although dominant interactions may vary by tumor type and stage, the review makes clear that CD147 coordinates both primary and metastatic TME.

In this context, Feigelman et al. showed that soluble EMMPRIN released by primary breast tumors promotes lung PMN formation. Rising with tumor burden, circulating EMMPRIN remodels the lung microenvironment by enhancing angiogenesis, neutrophil recruitment, fibroblast activation, and ECM re-organization. These effects, mediated through STAT3 and ERK1/2 signaling, identify soluble EMMPRIN as a systemic driver of PMN formation.

## 6 Conclusion

Taken together, the articles in this Research Topic underscore that the PMN and MN are shaped by dynamic interactions among tumor, stromal, and immune cells, the ECM, and cytokine/chemokine networks. These interactions vary by tumor type, organ site, and disease stage and influence metastatic seeding, dormancy, immune escape, and therapeutic response. Defining this network more precisely will be key to identifying therapeutic vulnerabilities and advancing mechanism-based combination therapies against metastasis.
